# Knowledge, perceptions, and feelings associated with Alzheimer’s disease and related dementias: a qualitative study among middle-aged latinas residing in an underserved agricultural community in California

**DOI:** 10.1186/s12889-024-20195-4

**Published:** 2024-10-17

**Authors:** Elizabeth Ambriz, Camila De Pierola, Morga C. Norma, Lucia Calderon, Katherine Kogut, Julianna Deardorff, Jacqueline M. Torres

**Affiliations:** 1 Department of Epidemiology & Biostatistics, University of California San Francisco, San Francisco, CA United States of America; 2 School of Public Health, University of California Berkeley, Berkeley, CA United States of America; 3 Center for Environmental Research and Community Health (CERCH), University of California Berkeley, Berkeley, CA United States of America

**Keywords:** Alzheimer’s, Dementia, Perceptions, Knowledge, Feelings, Agricultural, Qualitative research, Prevention, Health belief model, Social determinants, Middle age

## Abstract

**Background:**

Middle age is increasingly acknowledged as a critical window for prevention of Alzheimer’s disease and related dementia (ADRD) since research has shown that AD develops in the course of 20–30 years (1) but we know very little about middle-aged individuals’ perspectives on ADRD. Knowledge gaps are particularly large for Latinas living in regions typically underrepresented in ADRD research, such as rural and/or agricultural regions. This is important given that over the next 40 years Latinos are projected to have the largest increase in ADRD cases in the U.S. Therefore, this study aims to assess knowledge, perceptions, and feelings associated with ADRD among a sample of middle-age, Spanish-speaking Latina women.

**Method:**

Using qualitative methods involving semi-structured interviews, we examined knowledge, perceptions, and feelings associated with ADRD among a subsample of the Center for Health Assessment of Mothers and Children of Salinas (CHAMACOS) study. Participants are Latina women residing in an underserved agricultural community entering mid-life (mean = 46.5 years old). Interviews were conducted with 20 women and data was analyzed with inductive thematic content analysis.

**Results:**

We identified themes regarding perceptions, knowledge, and feelings. First, participants perceive ADRD as involving (1) Loss of memory, (2) Getting lost; (3) Losing the person they once were. With regard to knowledge about ADRD, participants reported: (1) Some knowledge about protective and risk factors for ADRD, (2) No awareness of the links between cardiovascular risk factors and ADRD; (3) A desire to learn prevention methods alongside signs and symptoms of ADRD. Themes related to feelings about ADRD were: (1) Fear of developing ADRD and not being aware of reality or who they are; (2) Worry about losing relationships with loved ones and caretaking if diagnosed with ADRD; (3) Sadness about forgetting one’s family and depending on others if diagnosed with ADRD.

**Conclusion:**

The knowledge gaps and negative feelings associated with ADRD highlighted in this study underscore the need for ADRD interventions to include CVD prevention, particularly for mid-life Latino populations residing in rural regions.

**Supplementary Information:**

The online version contains supplementary material available at 10.1186/s12889-024-20195-4.

## Background

By 2060, it is estimated that Latinos will represent nearly one-third of Americans and the Latino elderly population over 65 will nearly quadruple [1]. Over the next 40 years, Latinos are also projected to have the largest increase in Alzheimer’s disease and related dementia (ADRD) cases [1, 9]. It is estimated that at least one third of dementias are preventable and the following modifiable factors increase the risk: type 2 diabetes, smoking, mid-life hypertension, mid-life obesity, physical inactivity, depression, and low educational attainment [13, 14]. Cardiovascular risk factors, including high cholesterol, high blood pressure, obesity, smoking, and diabetes, are significant correlates of mild cognitive impairment and are highly prevalent among US Latinos [9]. Existing literature has supported the relationship between ADRD and cardiovascular risk factors among Latinos, as one study of 6,000 diverse Latinos revealed an association between the development of diabetes, mild cognitive impairment (MCI), and dementia [10]. Furthermore, US Latinos have been disproportionately affected by lower educational attainment, higher uninsurance rates, and earlier onset of Alzheimer’s when compared to non-Hispanic whites [2, 3]. A significant proportion of ADRD cases among Latinos could be preventable by addressing modifiable risk factors and conducting research to inform tailored and culturally adapted prevention and intervention strategies.

ADRD prevention and intervention efforts among Latinos are needed. However, there is a need to understand Latinos’ knowledge, perceptions, and feelings about ADRD to inform these efforts; very few studies have focused on this topic. In a notable exception, a qualitative study of knowledge and perceptions of Alzheimer’s among Mexican and Puerto Rican participants between 40 and 60 years of age reported a strong desire from both groups to have more education and community involvement with AD such as support groups or informational sessions in accessible locations like churches or schools [4]. Other studies have described that Latino adults in midlife are more likely to believe that ADRD is a normal part of aging or that they will not live long enough to experience such diseases [5, 6]. ADRD literacy gaps are concerning due to the existing mistrust and fear prevalent among the Latino community regarding research participation and healthcare utilization [7]. Moreover, one multicultural study in Massachusetts revealed that Latino dementia caregivers prioritize family care, take initiative to learn from Spanish-speaking providers, and worry about acculturation in terms of their children adopting the practice of institutionalizing elders [8].

While these studies provide crucial insights, several limitations remain, particularly, the existing literature has reported substantial heterogeneity among Latino subgroups, such that a focus on specific communities is imperative [4]. First, studies on this topic have primarily included participants over 60 years of age. However, there is growing recognition that middle age is a critical window for ADRD prevention, such that understanding perspectives on ADRD at this point in the lifecourse may be imperative. Second, most studies on knowledge, perceptions, and feelings about ADRD among Latinos have focused on geographically urban populations. Knowledge gaps may be particularly large for Latinos living in regions typically underrepresented in ADRD research, such as rural and/or agricultural regions. A focus on Latinos in rural and agricultural areas is necessary due to the shortage of accessible health resources, differences in educational and economic opportunities, and environmental conditions as compared to urban or suburban settings [11, 12]. To our knowledge, there is no substantial research focusing on the knowledge, perceptions, and feelings about ADRD among middle-aged Latinos in rural areas.

The present study addresses these knowledge gaps using qualitative methods to study knowledge, perceptions, and feelings associated with ADRD among a sample of middle-age, Spanish-speaking Mexican women living in an agricultural community. Our goal is to provide insights to inform culturally adapted prevention and intervention strategies designed to address modifiable risk factors among this population.

## Methods

### Study participants

Persons engaging in this qualitative study were participants in the ongoing Center for Health Assessment of Mothers and Children of Salinas (CHAMACOS) Study. The CHAMACOS Study includes women residing in a farmworker community in California (women’s children were also included in the broader study, but not in this qualitative research); the original participants were recruited in 1999/2000 and an additional group of demographically similar women and children were recruited in 2009. Follow-up visits for quantitative research have been conducted approximately every two years, with additional quantitative data collection done related to the impacts of the COVID-19 pandemic. Additional details about the CHAMACOS study have been published elsewhere [17].

As of 2021, a total of 594 maternal participants remained in the CHAMACOS cohort. While CHAMACOS mothers were in early adulthood at the start of the study, they are now primarily in mid-life (mean = 47). The Institutional Review Board (IRB) at the University of California, San Francisco (UCSF) approved this qualitative study (21-34842). CHAMACOS mothers were eligible for our qualitative study if they spoke Spanish (88% of the cohort) and lived in or near the Salinas Valley; all participants were 18 years or older.

We recruited participants in two ways. First, field staff distributed recruitment postcards to CHAMACOS mothers attending in person visits. The postcard invited women to contact the first author or share their contact information if they were interested in participating. Second, we sent a recruitment text message to a list of all potentially eligible CHAMACOS mothers who had participated in any recent aspect of CHAMACOS research (*n* = 467). If a CHAMACOS mother requested to be contacted or replied that they were interested in participating (*n* = 202), the research team followed up with a phone call to schedule a semi-structured interview (*n* = 32). Of those contacted via phone, a subset of (*n* = 12) were not available to participate or were not reachable. Data were collected from (*n* = 20) participants, at which point saturation was reached.

## Data Collection and methods

## Methods

Semi-structured interviews were conducted in Spanish by the first author, who is a bilingual and bicultural Latina researcher. Semi-structured interviews were professionally transcribed. The first and second authors checked transcripts for accuracy and then analyzed the Spanish transcripts. After the analysis, the first author translated relevant quotes to English during the manuscript writing process. In line with transparency and reflexivity in qualitative research [2, 3], the first author’s positionality as a Mexican immigrant researcher and daughter of farmworkers in the Salinas Valley, where this study was conducted, influenced the research process, the questions she asked, the data collection, and interpretation of data. However, she kept a memo to reflect throughout the research process.

Interviews lasted on average about 60 min and were audio recorded and professionally transcribed. The design of the interviews was informed by the Health Belief Model. Of relevance to the study are four factors of the model that are theorized to motivate actions and attitudes regarding one’s health: perceived susceptibility to the disease, perceived severity of the disease, perceived benefits of an action, and perceived barriers to taking action [15, 16]. We included questions related to these four factors including asking about knowledge about ADRD and prevention, perceptions of risk and protective factors, and feelings about ADRD. Semi-structured interviews were held in person in the CHAMACOS field office or virtually via the preferred video call platform for participants including Zoom, FaceTime, WhatsApp, or Messenger. Participants received a $75 gift card for their time. Semi-structured interviews were based on an interview guide that started with probing participants’ awareness, perceptions, and feelings related to ADRD (Appendix A). Informed written consent was obtained from all participants. Appendix B describes the processes the CHAMACOS team used to enhance trust between research participants and the research team.

## Analysis

Transcripts of semi-structured interviews were entered into MAXQDA software. The first author and an independent coder analyzed the transcripts using both deductive and inductive thematic content analysis [18, 19]. Transcripts were analyzed line by line to examine the knowledge, perceptions, and feelings associated with ADRD among middle-aged Latina women [20]. First, we focused on identifying subthemes under the deductive themes of knowledge, perception, and feelings. Next, we compared narratives from each semi-structured interview to assess whether they corroborated, negated, or expanded one another, which adds depth to the analysis [21]. For consistency and transparency, we used collaborative coding and followed the Consolidated Framework for Reporting Qualitative Research [22].

## Results

Twenty semi-structured interviews with middle-aged Latina women were conducted from November 2021 to January 2022. Demographic characteristics of the participants are illustrated in Table 1. Participant ages ranged from 39 to 59 (mean 46.5, standard deviation 5.7) and all participants preferred Spanish as their main language of communication. Almost half (45%) of participants were employed in an agricultural setting, 30% were unemployed, and 25% worked in a non-agricultural occupation.

**Table 1:** Demographic characteristics of middle-aged Latina women in a qualitative study of ADRD knowledge, perceptions and feelings (*n* = 20)

	*n* (%)
**Gender**	
Female	20 (100)
**Ethnicity**	
Mexican	20 (100)
**Age Category**	
30–39	2 (10)
40–49	14 (70)
50–59	4 (20)
**Preferred Language**	
Spanish	20 (100)
**Employment**	
Unemployed	6 (30)
Agriculture	9 (45)
Non-agriculture	5 (25)

Various subthemes identified under (1) perceptions, (2) knowledge, and (3) feelings related to ADRD. The sub themes are further described in the next sections and are presented in Table 2.

**Table 2:** Themes with corresponding exemplar quotes

**Perceptions**	
**Loss of memory**	“Well of Alzheimer’s, what I have heard is that they lose the ability to remember, and to remember how to do basic things like clean themselves, go to the bathroom, eat by themselves” (participant in late 30s)
**Getting lost**	“I have heard that they forget everything and, for example, if they are in the street, they get lost. They don’t even know where they are going” (participant in late 40s)
**Losing the person they once were**	“… I think that having that illness, you’re like a baby that will take up full attention, because I have also heard that like a foolish baby, you want to do things that you know you can’t” (participant in mid 40s)
**Knowledge of Risk factors**	
**Pesticide exposure**	“We live close to the fields, to the airplanes that go spraying pesticides. And also, I think that those illnesses come from there too” (participant in early 50s)
**Genetics**	“Well so, the risk [of ADRD] may increase by inheritance, perhaps by inheritance and well and the rest…” (participant in late 40s)
**Smoking and Alcohol**	“I think like those that smoke a lot, those that drink a lot, and a drink lot of coffee are at higher risk” (participant in late 30s)
**Over-worrying**	“Yes, when you’re living something very stressful, I don’t know, in your house, your relationship, problems with the children, with the husband, all that I think is very stressful and you work your mind too much in things that, that are making you think and think things” (participant in early 40s)
**Not Aware of link between CVD and ADRD**	“Well, that is new information, honestly, I hadn’t heard of that, but, well yes, generally, the illnesses almost go, like we say, one behind the other, even hand in hand” (participant in late 30s)
**Want to learn more about prevention**	“Well I would like to learn how to deal with people that have that illness, how to help them and another to prevent for oneself, in any case that you might have that illness, because you’re okay right now but you never know later on” (participant in late 40s)
**Knowledge of Protective factors**	
**Diet**	“How can I say, we like to eat more meat, instead of more vegetables. However, it is proven 100% that vegetables will keep you healthier.” (participant in early 40s)
**Exercise**	“Keep busy, because I already tell my husband, he always wants, strong because the body gets weaker and really if you don’t do an exercise, it weakens faster” (participant in early 40s)
**Mental Exercise**	“… for your brain, reading, learning new things and always keeping the mind busy to prevent, more than anything” (participant in early 40s)
**Feelings**	
**Fear**	“I would be afraid because like I’m telling you, if I were to forget things, if I were to get lost, I don’t even know in whose hands one is going to end up. That would be my fear” (participant in early 40s)
**Worry**	“Well it worries me or if someday it will get to me or something, not recognizing my children or my grandchildren, is what worries someone. Or that one would be in their home and leave and get lost. All that” (participant in late 50s)
**Sadness**	“Well yes, a bit of sadness because you imagine that you get that, you won’t remember your family, your children; if you get lost you have to depend on someone like a child, I think” (participant in early 40s)

### Perceptions of ADRD

Three sub themes were identified within the greater theme of perceptions of ADRD. When discussing their beliefs about the ways in which ADRD manifest in people, participants commonly mentioned loss of memory, physically getting lost, and loss of the person they once were.

## Loss of memory

ADRD were most notably perceived by all participants as involving memory decline, specifically related to memory of oneself, loved ones, and everyday tasks. Participants explicitly stated that one of the first signs of ADRD was forgetfulness. They described the potential loss of memory related to one’s self, as not knowing who they are. This theme is summarized by one participant stating, “… they say that it’s [ADRD] ugly because, well, you don’t know who you are or anything, and that’s how you forget things, sometimes like that” (participant in early 40s).

Loss of memory was also discussed in reference to one’s family and loved ones, citing that those with ADRD do not recognize their children and may confuse strangers for their loved ones. One participant illustrated this perception when recalling that “…I have just heard that they don’t remember things, they get lost, well sometimes don’t recognize their children, they look at other things, look at other people that are them, but in reality, aren’t them [their children]” (participant in late 40s). Lastly, cognitive decline was viewed by participants in terms of forgetting basic, everyday tasks such as using the restroom and eating, as detailed by one participant, “Well of Alzheimer’s, what I have heard is that they lose the ability to remember, and to remember how to do basic things like clean themselves, go to the bathroom, eat by themselves” (participant in late 30s). Overall, loss of memory in various contexts was a prominent indicator of ADRD among participants.

## Getting lost

Participants believed that the severity of the memory loss results in people forgetting how to return home when going out. Many participants expressed how the cognitive decline that is characteristic of ADRD can disorient a person to the point of getting physically lost. One participant describes a television segment regarding a woman with dementia when noting, “I think that in *La Rosa de Guadalupe* one time I saw a case of a person, a woman I think had that, and sometimes she left her home and would not return, she would get lost, like I tell you, they get lost, and there was everyone looking for them” (participant in early 40s).

Participants conveyed their perception that people with ADRD can go days without being found when lost because they may continue walking, but their destination is continuously forgotten. This phenomenon is stated by one participant, “Yes and well they take days in finding them because they don’t know where they are, and they walk and walk and don’t even know where to arrive” (participant in late 40s). The lack of control of one’s memory for a successful return is further supported by one participant adding, “I have heard that they forget everything and, for example, if they are in the street, they get lost. They don’t even know where they are going” (participant in late 40s). The inability to return home and continuous disorientation were frequently discussed by participants.

### Losing the person they once were

Equally as common as the theme of *getting lost* was the perception of losing the person they once were. Losing the person they once were was described as a return to the stage of infancy in which continued care is needed by others. One participant details the need for attention and monitoring of those with ADRD by explaining that “… I think that having that illness, you’re like a baby that will take up full attention, because I have also heard that like a foolish baby, you want to do things that you know you can’t” (participant in mid-40s). This theme was also described as a loss of one’s identity in which people with ADRD are viewed as a vessel for the disease rather than their personhood, as illustrated by two participants “I mean, well that they don’t know who the person is, I mean, that they don’t have knowledge of – or that you talk to them and no, they just look at you” (participant in early 40s) and “… No we don’t share much about the topic, only it was that she no longer understands, she no longer listens, she is already in another world…” (participant in late 40s). Additionally, the loss of humanity for participants includes the need for assistance with physical movement and functions, as listed by one participant stating, “… how to help them walk. How to help guide them, how to help them get up. I don’t know, many things come to my mind” (participant in late 30s). According to participants, the loss of the person they once were presents as a loss of independence and ability, relying on others for guidance and care.

### Knowledge of ADRD

Existing knowledge of ADRD among participants was divided into three categories including: (1) Some knowledge about protective and risk factors for ADRD, with pesticide exposure, genetics, smoking, alcohol, and over worrying as sub-themes related to perceived risk factors and diet, physical, and mental exercise as sub-themes related to perceived protective factors; (2) No awareness of the links between cardiovascular risk factors and ADRD; (3) A desire to learn prevention methods alongside signs and symptoms of ADRD.

### Pesticide exposure, Genetics, Smoking, Alcohol, and over-worrying as risk factors

ADRD risk factors were of high concern among participants; they cited pesticide exposure, genetics, smoking, alcohol, and over-worrying as potential ADRD risk factors. Some women discussed their geographical proximity to the fields and occupation in agriculture where pesticides are prominent, expressing their knowledge that these chemicals increase risk for ADRD and other diseases. One participant stated, “we live close to the fields, to the airplanes that go spraying pesticides. And also, I think that those illnesses [ADRD] come from there too” (participant in early 50s). Genetics were also commonly voiced in terms of ADRD being hereditary diseases, specified by one participant as, “Well so, the risk that it may increase is by inheritance, perhaps by inheritance and well and the rest…” (participant in late 40s). There was general knowledge of modifiable risk factors such as smoking and alcohol use. Others also noted risk factors that are not well-established (e.g. coffee use). One participant stated, “I think those that smoke a lot, those that drink a lot, and drink a lot of coffee are at higher risk” (participant in late 30s).

Over-worrying and overthinking were often discussed in conjunction with stress and said to be attributed to one’s family dynamics, work, and mental attitude, as illustrated by, “Yes, when you live in, in something very stressful, I don’t know, in your house, your relationship, problems with the children, with the husband, all that I think is very stressful and you work your mind too much in things that, that are making you think and think things” (participant in early 40s). Specifically, participants mentioned one ‘s outlook towards the future and life as potential contributors to ADRD risk, as supported by one participant who stated “It could also be the way of life we lead, our way of thinking. Sometimes we think about what will not happen, about what will happen, we think about the future instead of living in the present and we have many fears in our head” (participant in late 40s). Although over-worrying and overthinking have not been identified as risk factors for ADRD in the existing literature, these characteristics might influence stress pathways and health behaviors associated with ADRD such as smoking and alcohol consumption.

### Diet, Physical and Mental Exercise as protective factors

Participants reported substantial knowledge of ADRD protective factors including diet and physical and mental exercise. Physical exercise was mentioned most often among participants and consisted of various activities including walking, dancing, and working. The importance of keeping the body active in order to prevent debilitation was illustrated by one participant, “Keep busy, because I already tell my husband, if we want to stay strong, we need to exercise because the body gets weaker if you don’t do an exercise, it weakens faster” (participant in early 40s). Secondary was healthy eating in which meat was viewed as less healthy, while vegetables and fruits were frequently encouraged as shown by one participant’s lifestyle, “How can I say, we like to eat more meat, instead of more vegetables. However, it is proven 100% that vegetables will keep you healthier” (participant in early 40s). As well as physical exercise, mental exercise by keeping the mind active and occupied with activities such as reading, puzzles, and learning were identified as key protective factors as stated by one woman, “… for your brain, reading, learning new things and always keeping the mind busy to prevent, more than anything” (participant in early 40s). Participants demonstrated knowledge about keeping the mind and body both healthy and active to prevent the risk of ADRD.

### No awareness of the links between cardiovascular risk factors and ADRD

The majority (19/20) of participants were unaware of the relationship between ADRD and cardiovascular diseases. While knowledge gaps were prevalent, many women were not surprised about the association as shown by one participant, “Well that is new information, honestly, I hadn’t heard of that, but, well yes, generally, the illnesses almost go, like we say, one behind the other, even hand in hand” (participant in late 30s). Participants mentioned interest and concern about learning the relationship between both diseases, as demonstrated through two women, “All that? There it is, then it does interest me because I have high blood pressure” (participant in early 40s) and “That is new for me, I never thought that they would be related, but that is to worry about” (participant in early 40s). Overall, while unawareness was prevalent, participants exhibited an urge to learn, considering their own health concerns.

### Women desire to learn prevention methods alongside signs and symptoms of ADRD

Almost all the women explicitly expressed a desire to learn about ADRD signs and symptoms and strategies to prevent onset of disease. One participant summarizes this urgency to learn before treatment options are limited, “…knowing when the first symptoms are in order to prevent its progression, like you’re saying and if there are some ways to prevent it before it, before it gets to where there is no remedy” (participant in early 40s). Additionally, participants demonstrate empathy and wish to learn in order to help their loved ones and community, specifically to cope with people experiencing ADRD as stated by, “Well I would like to learn how to deal with people that have that illness, how to help them and another to prevent for oneself, in any case that you might have that illness, because you’re okay right now but you never know later on” (participant in late 40s). The importance of prevention knowledge is voiced through participants’ determination to practice strategies deemed preventative, as shown through one participant, “… if they tell me – it’s good to walk, for this; I’ll do it. Sometimes, although I may have a lot of laziness or the same depression, I get out. If there is a way to prevent Alzheimer’s disease…and dementia, well I would also do it, I would also do it for myself, for your own good” (participant in late 50s). Participants expressed wanting to expand their knowledge of ADRD for the sake of their own health and the health of others.

### Feelings related to ADRD

Participants voiced three primary feelings when discussing ADRD: (1) Fear of developing ADRD and not being aware of reality or who they are; (2) Worry about losing relationships with loved ones and caretaking if diagnosed with ADRD; (3) Sadness about forgetting one’s family and depending on others if diagnosed with ADRD.

### Fear of developing ADRD and not being aware of reality or who they are

Participants stated they felt a sense of fear about potentially developing ADRD. One participant communicated this feeling when stating, “Truthfully, it scares me that it will get to me” (participant in early 50s). Participants also discussed this feeling together with ADRD symptoms, citing their specific fear of disease outcomes as explained by one participant, “I would be afraid because like I’m telling you, if I were to forget things, if I were to get lost, I don’t even know in whose hands one is going to end up. That would be my fear” (participant in early 40s). Some participants also mentioned their fear in conjunction with forgetfulness they were currently experiencing, as illustrated by one woman, “The plain truth, for me it makes me tremble because for me, I continuously forget what I am going to do, what I said or what I planned with someone…” (participant in mid 40s). There was a consensus that the possibility of developing ADRD as well as experiencing symptoms of ADRD is frightening.

### Worry about losing relationships with loved ones and caretaking if diagnosed with ADRD

Participants across interviews indicated their worry of developing ADRD as well as certain symptoms that may arise. One participant voiced these worries and the possibility of not recognizing family members or getting lost, “Well it worries me or if someday it will get to me or something, not recognizing my children or my grandchildren, is what worries someone. Or that one would be in their home and leave and get lost. All that” (participant in late 50s). Additionally, one woman detailed her concern with treatment and survival following onset of ADRD, stating “Well the worry, I think is, insurance, how you’re going to cure yourself, how you’re going to help yourself and how you’re going to survive” (participant in late 50s). Participants also added that in their middle age, it is time to worry about ADRD as conveyed by one participant, “Yes, because I feel that for my age, I tell you that now, sometimes – I wouldn’t have thought it, but just how we are talking about it, it is now for worrying” (participant in mid 40s). Feelings of worry extended beyond the immediate disease, including concerns about caretaking and loved ones.

### Sadness about forgetting one’s family and depending on others if diagnosed with ADRD

The feeling of sadness was the most frequently mentioned sentiment among participants when discussing ADRD. Reasons for sadness extended through various aspects of ADRD, from symptoms of memory loss to patients’ family members coping with the disease. The feeling of sadness in regards to symptoms of ADRD and its emotional impact was demonstrated by two participants, “Oh, no, I think it is sad, forgetting everything and not knowing even where one is. How sad is that” (participant in late 40s) and “Well sadness, I feel like crying or – well that, sadness” (participant in early 40s). Participants expressed the sadness and pain that family members of someone with ADRD may experience as stated by one participant, “… seeing someone sick, I think that even oneself can get sick from the sadness of seeing a family member. Because I don’t think it won’t hurt someone to see a family member sick” (participant in mid 40s). Further, participants indicated feelings of sadness when discussing the potential to forget one’s family and depend on others due to ADRD progression as mentioned by one participant, “Well yes, a bit of sadness because you imagine that you get that, you won’t remember your family, your children; if you get lost you have to depend on someone like a child, I think” (participant in early 40s). This sentiment was eminent across study participants as they reflected on the various aspects of one’s life that ADRD effect.

## Discussion

This qualitative study analyzed the knowledge, perceptions, and feelings regarding ADRD among middle-aged Latina women in an agricultural region with an overall goal of informing future research and early prevention efforts. With the lack of diversity in ADRD research and underrepresentation of rural Latinos in particular, this study has provided significant information such as participants’ substantial knowledge of risk factors and protective factors, lack of ADRD literacy concerning CVD, a desire to learn about prevention, and feelings of sadness, worry, and fear in relation to ADRD. These results illuminate the substantial knowledge that this community holds as well as the need for preventative interventions that consider their feelings towards ADRD and concerns about social determinants.

### Knowledge of ADRD risk factors and protective factors

Participants in this study reported an understanding of various risk and protective factors for ADRD. Knowledge of risk factors include pesticide exposure and genetics as well as modifiable risk factors such as smoking, alcohol use, and over worrying. Many participants voiced a concern about the various pesticides and their health effects. This is perhaps due to their 10–20 years of experience in a research study focused on the health impacts of pesticide exposure and the fact that about half of our sample included agricultural workers. These worries are supported by evidence suggesting that extended exposure to pesticides could increase risk of developing ADRD, although more research on this topic is urgently needed [23]. Participants also expressed knowledge about healthy lifestyle behaviors that would serve as protective factors such as diet, physical exercise, and mental exercises – each of which theystate they try to incorporate in their lives. Participants were able to identify four core modifiable risk factors for ADRD throughout the study: smoking, alcohol, physical activity, and diet [24]. Even with existing literature calling for an increase of ADRD literacy among Latinos, our data show an understanding of many protective behaviors and risk factors, but desire to receive more detailed health information about ADRD including signs, symptoms, and ADRD-specific prevention methods to incorporate in their lifestyles.

Of the 20 participants, 19 did not know about the relationship between ADRD and CVD. This finding was striking considering the frequency in which participants mentioned their experience with cardiovascular problems such as diabetes and hypertension. Cardiovascular risk factors, including high cholesterol, high blood pressure, obesity, smoking, and diabetes, are significant correlates of mild cognitive impairment and ADRD while also disproportionately impacting U.S. Latinos [25, 26]. Additionally, the interaction between ADRD and cardiovascular disease has shown to present uniquely in the brains of Latinos compared to non-Hispanic whites. For example, a recent study of 423 autopsied brain tissue samples from individuals diagnosed with dementia concluded that dementia most commonly presented as a mixed diagnosis of Alzheimer’s and CVD in the brains of Latinos [27]. As a modifiable risk factor for ADRD, it is imperative that actions be taken to collaborate with community members to manage cardiovascular health and increase awareness of prevention. To our knowledge, there are no other studies measuring awareness of the relationship between CVD and ADRD among Latinos and these results show a need to increase ADRD literacy considering the high prevalence of CVD in this demographic.

While knowledge gaps were prevalent, participants also expressed an interest in learning. In particular, they desired to learn about ADRD prevention in terms of actions they could be taking to prevent the onset of ADRD, identifying signs and symptoms, and how to help someone who has ADRD. These participants exhibited a deep level of empathy, while they noted that they wanted to learn about preventative methods for their own health, they also would like to relay that information to other people in their social circles. Other scholars have identified the importance of outreach for Latinos through the utilization of existing community organizations such as churches and health programs to disseminate information about ADRD [9]. Furthermore, this study reveals the potential role of television shows such as La Rosa de Guadalupe in shaping knowledge, perceptions, and feelings about ADRD for participants since they mentioned a case of ADRD that was presented there. The show presents stories of people who have experienced miracles after praying to the Virgin of Guadalupe. The fact that participants mentioned the show elucidates the role of religious beliefs since in Mexico, where the majority of participants are from, the Virgin of Guadalupe is a central religious figure and people see her as a source of hope and strength. In addition, the current study highlights participants’ initiative to want to learn, although they also wished for more resources and information from professionals and trusted community partners. This perspective is evidenced in other studies revealing that Latinos believe ADRD care is important but do not know of treatments and services available or run into challenges finding care, often linguistically accessible care [9, 28]. Thus, barriers arise regarding actionable steps that Latinos can take to mitigate risk of developing ADRD for the sake of their health and the health of their community members.

#### Perceptions of ADRD

While there were perceptions of ADRD being a disease of memory loss, there was a lack of knowledge regarding the key symptoms of ADRD. Participants expressed that ADRD can be identified as one’s loss of memory, getting physically lost, and loss of one’s humanity. However, these signs and symptoms did not extend beyond the three mentioned, demonstrating a lack of knowledge regarding other components of ADRD that may help with prevention, such as changes in other aspects of cognition. This lack of awareness is supported by another qualitative study of ADRD literacy among middle-aged Latinos in Michigan that revealed knowledge gaps about the disease, its progression, and additional symptoms beyond memory loss [4]. Expanding awareness of ADRD signs and symptoms is crucial for early ADRD detection and treatment.

### Feelings related to ADRD

Lastly, results from this study indicate that participants are concerned about ADRD and expressed feelings of sadness, worry, and fear related to ADRD, even though they are in mid-life. Participants conveyed their fear of losing control due to the disease. They worried about how disease symptoms could affect their own lives, but they also worried about how it could affect their families. Barriers to health insurance and existing caregiving responsibilities within families heightened this worry. The feeling of sadness was the most popular sentiment mentioned due to the loss of memory and affect that ADRD may have on family members witnessing the disease take a toll on their loved one. These sentiments are important to understand when determining intervention strategies because Latinos have been shown to perceive personal beliefs, language proficiency, and economic status as the greatest barriers to healthcare [29]. Participants expressed serious concern and urgency about the impact of ADRD on their community and families, which may increase their receptiveness to educational initiatives designed to address modifiable risk factors. Understanding the feelings that community members experience and the factors shaping these sentiments can help inform tailored interventions to center their concerns.

### Implications of social determinants

It is critical to consider the social determinants of health such as the environment in which this community lives and works when addressing ADRD risk and informing intervention strategies. The manifestation of such social determinants is illustrated through the participants’ categorization of over thinking and over worrying as a risk factor, often citing the stress of work and home responsibilities. While some participants believed it was important to combat this stress by managing one’s outlook, the association of stress and ADRD described by participants is crucial for understanding how social determinants are affecting their daily lives. Studies have shown that social determinants including manual labor, higher levels of stress, and early-life adversity are associated with a higher risk of ADRD [30]. Latinos experience chronic stress related to social determinants, which are known to exacerbate the likelihood of developing chronic illnesses, namely cardiovascular disease and diabetes, which are risk factors for cognitive impairment and the development of dementia [30, 31, 32]. Participants’ unique identities as Mexican immigrant women and farmworkers shape their experiences and lived experience with pesticides in their environment, their stressful lifestyles balancing home and work responsibilities, and a few disclosing concerns about immigration status. Furthermore, their unique experiences as a marginalized group influence their perceptions, knowledge, and feelings about ADRD as illustrated in the theme of “over worrying” as a risk factor for ADRDs.

In line with the health belief model, which is theorized to motivate actions and attitudes regarding one’s health: participants in this study worry about developing ADRD themselves, perceived a high severity of the disease, and are interested in taking preventive action and expanding their knowledge on ADRD risk and protective factors [16]. For these reasons, it is important to design interventions to address modifiable risk factors for ADRD among Latinos, that are centered in their lived experience. In addition, this information regarding social determinants also highlights critical areas for future ADRD research, particularly quantitative studies on the effects of pesticide exposure on cognition. Expanding knowledge on the impact that social determinants have on ADRD, specifically among Latinos, would provide holistic insight to address ADRD inequities.

### Limitations

One limitation is that all of the women have participated in the CHAMACOS study for over 20 years, and they had exposure, through educational forums and newsletters, to the concept that pesticides impact a number of health outcomes. However, the study had not previously focused on or provided education about ADRD.

### Recommendations for future research

The knowledge gaps of CVD and ADRD highlighted in this study underscore the need for ADRD interventions to include CVD prevention, particularly for mid-life Latino populations residing in rural regions. Cardiovascular risk factors (CVRF) in early adulthood and midlife are modifiable targets to mitigate or reduce racial/ethnic disparities in cognitive impairment and dementia. Latinos have a high prevalence and mortality associated with CVRF which is associated with an increase in dementia risk [33]. Due to its potential to reduce ADRD risk, CVRF could be addressed with culturally informed interventions among this demographic.

Additionally, community-based research puts the community at the forefront and seeks to understand the needs and perceptions of the population to collaboratively tailor interventions. The intention of this study is to provide insight from this regionally-specific Mexican population and guide the focus of future research and health information for the community. With Latinos of Mexican origin being the largest Latino subgroup in the U.S., insight to perceptions, knowledge, and feelings of this specific ethnic group can demystify assumptions of homogenous beliefs among Latinos. As Latinos are expected to have the steepest increase in ADRD by 2060 and estimated to be 1.5 times more prone to develop Alzheimer’s than White non-Hispanics, increased engagement within this community is necessary now more than ever [29].

## Conclusion

In this qualitative study, middle-aged Latina women in an agricultural area hold some knowledge about ADRD protective and risk factors, and a desire to learn more about prevention and CVD as ADRD risk factors. Perceptions of ADRD exemplified the need to expand health literacy regarding signs and symptoms. By sharing feelings towards ADRD, participants provided insight to their concerns and the impact that ADRD have on them, their family, and their community. Overall, middle-aged Latinos should be prioritized in ADRD research and community engagements to disseminate knowledge regarding prevention strategies and implement tangible interventions. Addressing modifiable risk factors related to CVD is crucial as the burden of ADRD and CVD among Latinos in conjunction with social determinants impact health disparities that must be combated with urgency.

## Electronic supplementary material

Below is the link to the electronic supplementary material.

Supplementary Material 1

Supplementary Material 2

## Data Availability

The datasets used and/or analysed during the current study available from the corresponding author on reasonable request.

## Abbreviations

ADRD: Alzheimer’s Disease and Related Dementia
CVD: Cardiovascular Disease
CVRF: Cardiovascular Risk Factors
MCI: Mild Cognitive Impairment
CHAMACOS: Center for Health Assessment of Mothers and Children of Salinas Study
