# Supplementary material for: Knowledge, perceptions, and feelings associated with Alzheimer’s disease and related dementias: a qualitative study among middle-aged latinas residing in an underserved agricultural community in California
Source: BMC Public Health. 2024 Oct 17;24:2865. doi: 10.1186/s12889-024-20195-4 (PMC11488234; doi:10.1186/s12889-024-20195-4)
Supplement: Supplementary file 1 — Supplementary Material 1 [file 12889_2024_20195_MOESM1_ESM.docx]

**Appendix A. Semi-Structured Interview Guide**

**Aim 2. To assess knowledge, perceptions, and feelings about Alzheimer’s Disease and Related Dementias (ADRDs) and their prevention**

1. Is there someone in your life who is affected by Alzheimer’s or dementia? If yes, tell me more about your relationship with that person.
   1. How has their condition affected you and how does it make you feel?
2. What have you heard and learned about brain diseases such as Alzheimer’s and dementia?
   1. What kind of thoughts do Alzheimer’s and dementia evoke? Why?
3. How do you keep your brain healthy?
   1. Do you believe Alzheimer’s and dementia can be prevented (why/why not)? If yes, how?
   2. Is there anything one can do to reduce the risk of Alzheimer’s and dementia?
   3. What are some things one can do to prevent or delay the onset of Alzheimer’s and dementia?
   4. What motivates you or would motivate you towards prevention of Alzheimer’s and dementia?
   5. What stops you or would stop you from preventing Alzheimer’s and dementia?
4. What increases your risk for developing of Alzheimer’s and dementia?
   1. What are some behaviors or conditions that increase your risk for developing Alzheimer’s and dementia?
5. How do you feel that Alzheimer’s and dementia are related to illness in other parts of the body like CVD (diabetes, hypertension, obesity)? Do you think there is a connection between CVD and Alzheimer’s and dementia?

**Perceptions of and feelings associated with Alzheimer’s and dementia**

1. What kind of feelings do Alzheimer’s and dementia evoke? Why?
2. What scares/worries you about Alzheimer’s and dementia?
3. What is your likelihood of developing Alzheimer’s or dementia? (perceived susceptibility)
4. What would be concerning, make you worry in yourself and others?
