# Supplementary material for: Knowledge, perceptions, and feelings associated with Alzheimer’s disease and related dementias: a qualitative study among middle-aged latinas residing in an underserved agricultural community in California
Source: BMC Public Health. 2024 Oct 17;24:2865. doi: 10.1186/s12889-024-20195-4 (PMC11488234; doi:10.1186/s12889-024-20195-4)
Supplement: Supplementary file 2 — Supplementary Material 2 [file 12889_2024_20195_MOESM2_ESM.docx]

# HUMAN SUBJECTS

1. **Human Subjects involvement, characteristics, and design**

***Overview.*** This proposal aims to assess knowledge, perceptions, and feelings about Alzheimer’s Disease and Related Dementias (ADRDs) and their prevention among mid-life Latinas residing in rural communities.

***Participants.*** This research will leverage approximately 600 women who have participated in the ongoing CHAMACOS study. Nearly all CHAMACOS families are of Mexican origin, with 86% of women (mothers in CHAMACOS families) having emigrated from Mexico. Women enrolled in the study during one of two separate waves of recruitment: during their pregnancy in 1999-2000 (“CHAM1” wave) or in 2009-2011 when their children were 9 years old (“CHAM2” wave). Women were not selected on any health conditions, though all had given birth to at least one child. The majority of women are still working, with many doing physical labor in agricultural fields, and are in generally good health. However, many are overweight or obese, have high blood pressure, experience anxiety and/or depression, and have other health conditions common among U.S. women of this age. The study has collected data from these families prospectively since enrollment, and our proposed research includes new data collection from women at two time points – the 20-year CHAMACOS follow-up visit (2021, when women will be an average of 47, range: 36-66) and the 22-year follow-up (2023, when women will be an average of 49 years old, range: 38-68). This study expects to engage n=20 to participate the semi-structured interviews.

***Recruitment.*** Recruitment for proposed new data collection will be focused on women in the CHAMACOS cohort who reside in Salinas Valley. First, field staff will distribute recruitment postcards to CHAMACOS mothers attending in person visits. The postcard invited women to contact the first author or share their contact information if they are interested in participating. Second, we will send a recruitment text message to a list of all potentially eligible CHAMACOS mothers who had participated in any recent aspect of CHAMACOS research (n = 467). For women who are interested in participating, we will summarize the basic data collection activities to be completed during the interview, explain incentives, the location of study visits, and the transportation support we can provide. For those who express interest in the study, we will schedule an interview at our Salinas field office or at a convenient location that participants select. Full written informed consent for qualitative data collection from semi-structured interviews will be done before starting the interview.

- 1. ***Use of existing data.*** We will make use of **existing data** collected from CHAMACOS maternal participants under protocols approved by the UC Berkeley Committee for the Protection of Human Subjects, at visits conducted at all prior waves of collection. These data include:
     - Questionnaire data collected from women on a wide variety of topics at multiple time points, including sociodemographic information and life stressors.
  2. ***Proposed new data collection.*** We will also make use of **new qualitative data** to be collected from women as part of the proposed study.

Data collection activities will be conducted with assistance of Salinas-based research. All research staff have been interviewed, selected, trained by, and currently report to UC Berkeley investigators.

***Incentives.*** Participants will be compensated $75 for completion of the semi-structured interview. The compensation we propose is intended to reimburse women for their time and participation. We feel this amount adequately compensates participants for their time but is not high enough to coerce participation from individuals who prefer to decline.

1. ***Returning results.*** Participants will be informed of aggregate study results via a brief, bilingual newsletter distributed by mail and via our website upon completion of the data analyses planned in this proposal. We will also partner with the CHAMACOS study to host an in-person event to present the results.

# Potential risks to subjects.

- 1. The primary potential risks to participants are loss of time, loss of privacy, emotional distress, and breach of confidentiality, as summarized here.
     - ***Loss of time:*** Participants may find it inconvenient to travel to the study visit and spend time participating in the semi-structured interviews. The likelihood that at least some participants will feel inconvenienced by this time loss is high, but the anticipated seriousness is low.
     - ***Loss of privacy:*** Participants will be asked to answer questions about some private topics, including finances, social stressors, and depression. The likelihood that participants will lose some privacy is high, but the seriousness to participants is relatively low, given that participants have answered these types of questions for many years and know to anticipate them.
     - ***Emotional distress:*** Participants may experience embarrassment, shame, anxiety, or distress when asked sensitive questions about the topics outlined above. The likelihood that at least some participants will experience emotional discomfort is high, but because these experienced participants can anticipate that questions of this nature will be asked, we expect the seriousness to participants to be relatively low.
     - ***Breach of confidentiality:*** Accidental or purposeful breach of confidentiality (e.g. when we must report suspected abuse or risk of harm to self or others) could conceivably harm participants’ reputations and/or put them or family members at legal risk. The likelihood of a breach of this nature is very low, but the seriousness could be very high.

# Adequacy of protection against risks.

***Protections:*** We will take the following steps to minimize risk to participants:

- ***Loss of time***: We will clearly inform all participants of the interview length and anticipated round-trip travel time. We will work with individual participants to best accommodate their schedules.
- ***Loss of privacy***: We will remind participants about the voluntary nature of participation and information that they share, including a reminder that they may decline to answer any question(s) they wish.
- ***Consent procedures:*** Written consent will be obtained from all participants. This will take place in two stages. Before participants schedule their interview, they will receive a phone call in which we will briefly summarize the study purpose and visit activities. This preliminary contact allows potential participants time to reflect at home on whether they would like to participate. When participants arrive for their interview, they receive more complete information within the informed consent process. Participants will be invited to read along on their own print copy as they listen to the consent form read aloud to them in their language of choice (typically Spanish) by a Study Interviewer. All will be informed of the overarching aims of the study, the nature of the questions we will ask them, our confidentiality policies (emphasizing that we will never release data other than to approved researchers unless we suspect imminent risk of harm to self or others, and that their data are otherwise protected from forced disclosure by a Certificate of Confidentiality), and the voluntary nature of participation, including a reminder that they may decline to answer any question(s) they wish.
- ***Emergency and referral procedures.*** We do not anticipate adverse psychological effects to subjects, but do have emergency procedures in place to respond to psychiatric emergencies (e.g. suicidal ideation) detected in the course of our work with human subjects. One of our collaborators, licensed clinical psychologist Dr. Deardorff, trains staff in emergency response procedures and coordinates individual responses to such situations. Responses to such situations have included walking the participant to the emergency room (located on the same campus as our research office), and/or assisting participants in scheduling urgent appointments with local counsellors.
- ***Protection of vulnerable groups.*** The founding purpose of the CHAMACOS Study was to assess the impact of pesticide exposures in a population disproportionally exposed to pesticides: agricultural fieldworkers and their family members. This includes individuals with undocumented immigrant status and adults with limited literacy. In the U.S., Mexican- Americans and Mexican immigrants are overrepresented in the farmworker population, and many of the latter group are undocumented. Many migrant workers and family members have received minimal education, and this is reflected in limited literacy. We obtained a Certificate of Confidentiality for this study with the special vulnerability of our undocumented immigrant participants in mind. We have also crafted our consent procedures in response to the limited literacy of many adult participants. We write our consent materials as simply as possible, and require that our Study Interviewers read all consent materials aloud to adult participants.

1. ***CHAMACOS privacy and confidentiality considerations.*** We inform all participants during the consent procedure that we will not disclose personal information about them except in rare specific instances: specifically, when we suspect child abuse or neglect, elder abuse, or a risk of harm to self or others. We inform all participants in the consent process of these limits to confidentiality. We do not ask questions with responses that would mandate reporting. The most frequent circumstance we encounter that challenges maintenance of confidentiality is suicidal intention; in all instances to date, we have been able to work with the participant to help them seek help for themselves, including walking with them to the emergency room near our field office.

# Relationship between researcher and community

We are committed to Community Based Participatory Research (CBPR), and have developed a relationship of trust and collaboration with the Salinas Valley community in the 22 years since the CHAMACOS study was founded. We have established a diverse Community Advisory Board, which provides input on research endeavors and is informed of latest research findings prior to publication. We have held regular community forum events for CHAMACOS participants, which include an accessible summary of recent research findings, tips on how participants can limit their exposure to environmental chemicals, and learning activities for youth. For this proposal, we are planning to hold a community at the end of this study to share results with participant families and other interested community members.

# Potential benefits of the research to the participants and others

Historically, the main benefits of the research to CHAMACOS participants have been enhanced access to community resources, increased knowledge about social and environmental health issues, and feedback on potential health issues detected in our screenings. With regards to community resources, the Salinas-based field staff that facilitated all data collection through age 20 years was very well acquainted with health, counseling, and related resources in the Salinas Valley community, and readily provided participants with information tailored to their needs. In addition, we distributed community resource guides to all participants. Another benefit to participants has been increased knowledge about environmental health research and local environmental health issues (e.g., how one can be exposed to agricultural pesticides, and strategies to minimize these exposures). In addition, our proposed study has the potential to benefit participants who will be invited to a series of educational workshops on ADRD prevention organized in partnership with the Monterey Peninsula Alzheimer’s Association. These workshops are designed to support participants in making improvements in various risk and protective factors for ADRD. Participants can also take pride in their significant lifetime contribution to a scientific endeavor that receives frequent media coverage for its significant new research findings.

# Importance of the knowledge to be gained

The proposed research will help fill a critical gap and further inform the development, refinement, and testing of culturally adapted interventions to improve outcomes among mid-life women living in an underserved agricultural setting that has been underrepresented in aging research. The implications of this research are far-reaching. In so doing, it will identify targets for intervention adaptations and policy changes to prevent poor outcomes in mid-life. We feel that the potential risks of this research are reasonable relative to the importance of the knowledge to be gained. Many of our participants have noted that they are committed to the study because they are interested in learning how to improve their own health in the process and are invested in seeing our research questions answered.
